# Impact of COVID-19 on energy consumption in a residential complex in Hyderabad, India

**DOI:** 10.1186/s42162-022-00240-5

**Published:** 2022-12-21

**Authors:** Kuntal Chattopadhyay, Vishal Garg, Praveen Paruchuri, Jyotirmay Mathur, Srinivas Valluri

**Affiliations:** 1 Center for IT in Building Science, International Institute of Information Technology- Hyderabad, Hyderabad, 500032 India; 2 Machine Learning Lab, International Institute of Information Technology- Hyderabad, Hyderabad, 500032 India; 3 Centre for Energy and Environment, Malaviya National Institute of Technology Jaipur, Jaipur, 302017 India; 4 CEO- Synergy Infra Consultants (Pvt)Ltd, Hyderabad, 500082 India

**Keywords:** COVID-19, Residential energy consumption, Air conditioning energy consumption, Household electricity consumption, Case study, Data monitoring, India

## Abstract

When the Indian government declared the first lockdown on 25 March 2020 to control the increasing number of COVID-19 cases, people were forced to stay and work from home. The aim of this study is to quantify the impact of stay-at-home orders on residential Air Conditioning (AC) energy and household electricity consumption (excluding AC energy). This was done using monitored data from 380 homes in a group of five buildings in Hyderabad, India. We gathered AC energy and household electricity consumption data at a 30-min interval for each home individually in April 2019 and April 2020. Descriptive and inferential statistical analysis was done on this data. To offset the difference in temperatures for the month of April in 2019 and 2020, only those weekdays were selected where the average temperature in 2019 was same as the average temperature in 2020. The study establishes that the average number of hours the AC was used per day in each home increased in the range 4.90–7.45% depending on the temperature for the year 2020. Correspondingly, the overall AC consumption increased in the range 3.60–4.5%, however the daytime (8:00 AM to 8:00 PM) AC energy consumption increased in the range 22–26% and nighttime (8:00 PM to 8:00 AM) AC energy consumption decreased by 5–7% in the year 2020. The study showed a rise in household electricity consumption of about 15% for the entire day in the year 2020. The household electricity consumption increased during daytime by 22- 27.50% and 1.90- 6.6% during the nighttime. It was observed that the morning household electricity peak demand shifted from 7:00 AM in 2019 to 9:00 AM in 2020. Conversely, the evening peak demand shifted from 9:00 PM in 2019 to 7:00 PM in 2020. An additional peak was observed during afternoon hours in the lockdown.

## Introduction

Severe Acute Respiratory Syndrome Coronavirus 2 (SARS-CoV-2) is the primary cause of the pandemic that started in 2019. COVID-19 has significantly influenced life all over the world. It spread rapidly, infected around half a million people, and killed more than 20 thousand over the globe until 27 March 2020 (https://www.who.int/docs/default-source/coronaviruse/situation-reports/20200327-sitrep-67-covid-19.pdf). The impact of COVID-19 was devastating worldwide, in terms of the economy, health, social and emotional aspects. An unprecedented nation-wide lockdown brought a grinding halt to economic activities, adversely affecting the lives and livelihoods of thousands of daily wage workers. The economic impact was drastic, which led researchers to study transformation in every sector.

The first positive case of COVID-19 was reported in India on 27 January 2020 (Andrews et al. 2020). By mid-April 2020, India recorded around 12,370 COVID-19 cases and 423 deaths (https://www.worldometers.info/coronavirus/country/india/). Initially, many steps were taken to control the spread. The prime minister of India called for ‘Janata curfew’ on 22 March from 7 AM-9 PM, urging people to stay home except those working in essential services and enforcing public led social distancing interventions (https://www.who.int/india/emergencies/coronavirusdisease-(covid-19)/india-situation-report). However, the nation was still not able to completely control the situation. Finally, on 25 March 2020 the first lockdown for the entire nation was imposed to tackle the spread of the pandemic (https://www.timesnownews.com/india/article/march-25-2020-the-dayindia-went-into-nationwide-lockdown-to-tackle-coronavirus/736838). The focus was on the closure of all activities except essential services such as hospitals, telecom, pharmaceuticals, and provisional stores (https://www.mha.gov.in/sites/default/files/Guidelines_0.pdf). People saw technology and internet-based services as a major source to communicate, interact, and perform their work from home (https://zeenews.india.com/technology/how-technology-changed-lives-during-covid19-lockdown-2349849.html).

Above all, these restrictions affected production activities and individual ways of living, leading to noticeable changes in energy consumption. According to the reports, more than 80% of the workspaces were closed completely or partially worldwide (Chen et al. 2020). Globally, the energy demand reduced by 3.8% within the first three months of lockdown (https://www.iea.org/reports/global-energy-review2020), although there was an increase in the energy demand for residential buildings (https://www.tdworld.com/distributed-energy-resources/demand-sidemanagement/article/21128542/covid19-is-changing-residential-electricity-demand). UK, Spain, France, and India saw their consumption decreased by almost 15% during lockdown periods. The electricity demand of Italy also decreased by 35%. In China, energy utilization dropped by 6.5% in the first quarter and (https://www.iea.org/reports/covid-19-impacton-electricity). Studies (https://www.iea.org/reports/covid-19-impacton-electricity; Ashkanani et al. 2022; https://www.iea.org/reports/portugal-2021) have shown that overall country level consumption decreased during the pandemic. A case study done on Turkey by Yukesltan et al. (2022), using a modulated Fourier series expansion on overall electricity consumption, forecasted that the demand will decrease by up to 12% according to the level of restrictions imposed. Furthermore, Wen et al. (2022) predicted a decline of 12% electricity demand for New Zealand using the auto-regressive-moving-average model. Sánchez-Úbeda et al. (2022) analysed electricity demand of Latin America and Caribbean countries (Peru, Bolivia, Costa Rica, Brazil, Guatemala, Mexico, Dominican Republic, Argentina, Chile, and Uruguay), and found a decrease of 30% for Peru and Bolivia. While a decrease of 6% was observed for Chile and Uruguay, remaining countries had a reduction by 11 to 17%. Alavi et al. (2022) observed a reduction of electricity consumption for Bangladesh by 50%. In addition, they developed a neural- network based prediction model, which can predict electricity consumption for Bangladesh if a lockdown is announced in future.

Several studies (Edomah and Ndulue 2020; Burleyson et al. 2021) have shown that there was an energy shift from commercial and industrial buildings to residential buildings. Sanchez-lopez et al. (2022) conducted a similar kind of study on the impact of electricity demand in Chile, using data obtained from 230 thousand smart meters. They noticed a rise of 17% in June, when compared to the same month of 2019. Conversely, the industrial electricity demand was reduced by 75%. Krarti and Aldubyan (2021), through their review study, observed the post-pandemic effect on energy consumption of the residential sector as a function of normalized weather time series data. A rise of 11–32% for few countries (Australia, UK, and the USA) during complete lockdown was observed. This massive shift of energy demand from industrial and commercial buildings towards residential buildings provides an idea of the impact of lockdown during the pandemic.

Several researchers have studied the impact of COVID-19 on residential energy demand. A case study done by Aldubayan and Krarti (2022) on residential buildings of Saudi Arabia evaluates the short-term impact of the lockdown. Their study finds a surge of 25.2% in electricity consumption during the lockdown period. Later, when residential building stock models were used, using normalised weather conditions, they found an increase of 16% in housing energy compared to the year 2019. Most of the increase was due to a significantly higher usage of lighting, appliances, and air-conditioning associated with increased occupancy levels during daytime hours. Further, using the same validated residential building model they forecasted that the overall energy consumption can increase upto 13.5% if stay-home living continues compared to the year 2019. Utilizing the information gathered from a study in Ireland, the general increase in energy utilization for houses has been assessed to range between 11 and 20% during the lockdown time frame, with even higher increments happening during 9 AM to 5 PM on working days (https://www.savills.us/insight-and-opinion/savills-news/299070/covid-19-restrictions-changing-the-daily-patterns-of-energy-consumption).

Meanwhile, Abdeen et al. (2021) explored the household hourly electricity consumption in Canada. Using measured electricity consumption data from 500 homes in Ottawa, they observed that daily electricity consumption increased by 12% relative to the non-COVID year. A similar kind of study done by Qarnain et al. (2020) found 34 factors responsible for driving more energy consumption in the residential sector of India, finally concluding that energy was consumed more intensely in pandemic times than in pre-pandemic times. An increase of 12% residential energy consumption after a few weeks of lockdown was reported by Austin Energy (https://www.statesman.com/story/news/local/flashbriefing/2020/03/25/austins-coronavirus-stay-home-order-could-swell-utilitybills/1459345007/). Besides, evaluating the high- frequency electricity data from 491 houses and interviews on household energy consumption with 17 families in Queensland, Snow et al. (2020) compared energy use before and during COVID-19 lockdown. They estimated the key factors responsible for household electricity consumption, and cooking and digital devices were the major contributors. Rouleau and Gosselin (2021) recently studied a 40-dwelling social housing building located in Canada and noticed occupants were using more electricity from 9:00 AM to 5:00 PM. An increase of 46% was seen relative to the same month in pre-COVID time. A study done by Bielecki et al. (2021) on data obtained from 7000 flats, observed that while energy consumption has increased, there was no change in the average daily peak of these houses. Simulation studies were conducted by few researchers (Li et al. 2021; Ding et al. 2021; Ku et al. 2022) to estimate the long-term impact of the lockdown on residential electrical demand. They estimated an increase in the electricity consumption range of 13–27%.

There were also few studies conducted on the variation in energy patterns and the increase of peak demand for residential buildings. The studies also tried to find which devices contributed to the increase in energy consumption. Aldubyan and Krarti (2022) further demonstrated the hike of peak demand by 15 to 20% in post-COVID as compared to pre-COVID. Li et al. (2021) through their simulation results also predicted a hike of 35–53% in residential hourly peak demand between 12 and 5PM. An energy use survey was conducted by Huebner et al. (2021) in the UK for 1016 participants during the first lockdown in March 2020. The survey data concluded that the electricity demand was more during the day and Italy showed an increased usage of cooking appliances, television and computers. Surahman et al. (2022) investigated household energy consumption of urban residential buildings in major cities of Indonesia during COVID-19 pandemic. Statistical analysis performed on the survey results received from 311 residents concluded that the average annual energy consumption of samples taken was larger by 3 GJ during pandemic. The increase was majorly due to excessive use of AC and cooking appliances. A study done by Chinthavali et al. (2022) noticed a significant change in the pattern of electricity load in residential buildings during weekdays in lockdown. They also confirmed that HVAC and water heaters are the largest consumers of electricity in residential homes. Further, Kranti and Aldubyan (2021) added that most of the energy was consumed by HVAC. Kawka and Cetin (2021) compared the HVAC loads, non-HVAC loads and overall loads of 225 housing units located in Texas, for the duration 2018–2020 and concluded that maximum energy consumption in non-HVAC residential buildings occurred between 10 AM-5 PM while HVAC loads also increased for the lockdown period.

According to data published by Times of India (TOI), India also witnessed a decline in energy consumption of 25% in the last week of March 2020, which was more than the decline that occurred in the US and Europe (https://www.indiatoday.in/india/story/india-s-decline-in-electricityconsumption-due-to-lockdown-more-severe-than-us-and-eu-1666444-2020-04-13). A study done by the Prayas (energy group) (https://www.prayaspune.org/peg/blogs/household-electricity-consumption-in-indiaduring-the-covid-19-lockdown-insights-from-metering-data.html) on minute-wise load and voltage data of 81 households located in Uttar Pradesh and Maharashtra from 4 March to 5 May 2020, observed that the daily average household energy consumption increased by 26% in the lockdown period as compared to the pre-lockdown period. The finding was that among all household equipment, Heating, Ventilation, and Air Conditioning (HVAC) and water heaters consumed the most energy. They analysed houses with and without AC and concluded that AC-homes consumption increased by 45–60% while non-AC homes energy consumption increased by 22% as compared to pre-COVID times. In their study, they showed that residential energy demand depends upon the size of the home, occupancy, climate of the place, and the location. Apart from this small-scale analysis there is a lack of similar studies in the Indian context.

The objective of our study is to evaluate the impact of COVID-19 on the AC energy and household electricity (excluding AC energy) consumption independently. AC energy indicates the amount of energy consumed by AC and household electricity indicates the total energy consumed by remaining electrical appliances excluding AC. Further, the following hypotheses were made, and tested:

- Working from home during weekdays would lead to an increase in energy consumption since occupants may cook lunch, use AC and other electrical devices during the day.
- The energy consumption during night-time is expected to reduce due to the pre-cooling effect when ACs are operated during daytime.
- There may be a delay in the morning peak since people may wake up late as they don’t need to commute due to the work from home.

The results were also checked to confirm whether these points are statistically significant or not. The paper is structured into five sections: “Methodology” section provides the details of study area and selection of month for the analysis, followed by details of the buildings selected for the data collection and data pre-processing. “Results” section provides the results obtained from the analysis, comparison between non-COVID and COVID year for the overall AC energy consumption and household electricity consumption of the homes. The operating hours for the AC were also compared for both the years. Further insights are provided for the daytime and nighttime AC energy consumption, operational hours, and household electricity consumption for the same, followed by statistical tests for significance and frequency distribution of the data. A comparison with the current literature was done in “Discussion” section. Finally, the paper is concluded in the last section along with the test results.

## Methodology

### Study area and duration

With a population of over 1.4 billion, India is the second most populated country and the seventh-largest country by area. Located in the south of Asia (https://www.ibef.org/economy/indiasnapshot/aboutindia-at-a-glance), India’s climate can be classified as hot tropical (https://www.weatheronline.co.uk/reports/climate/India.htm). The temperature ranges from 45 °C in summer to 5 °C during winter in various parts of the country. Summer in India begins from March and continues till June (https://www.indiaonlinepages.com/weather/summers-in-india.html).

**Fig. 1:**
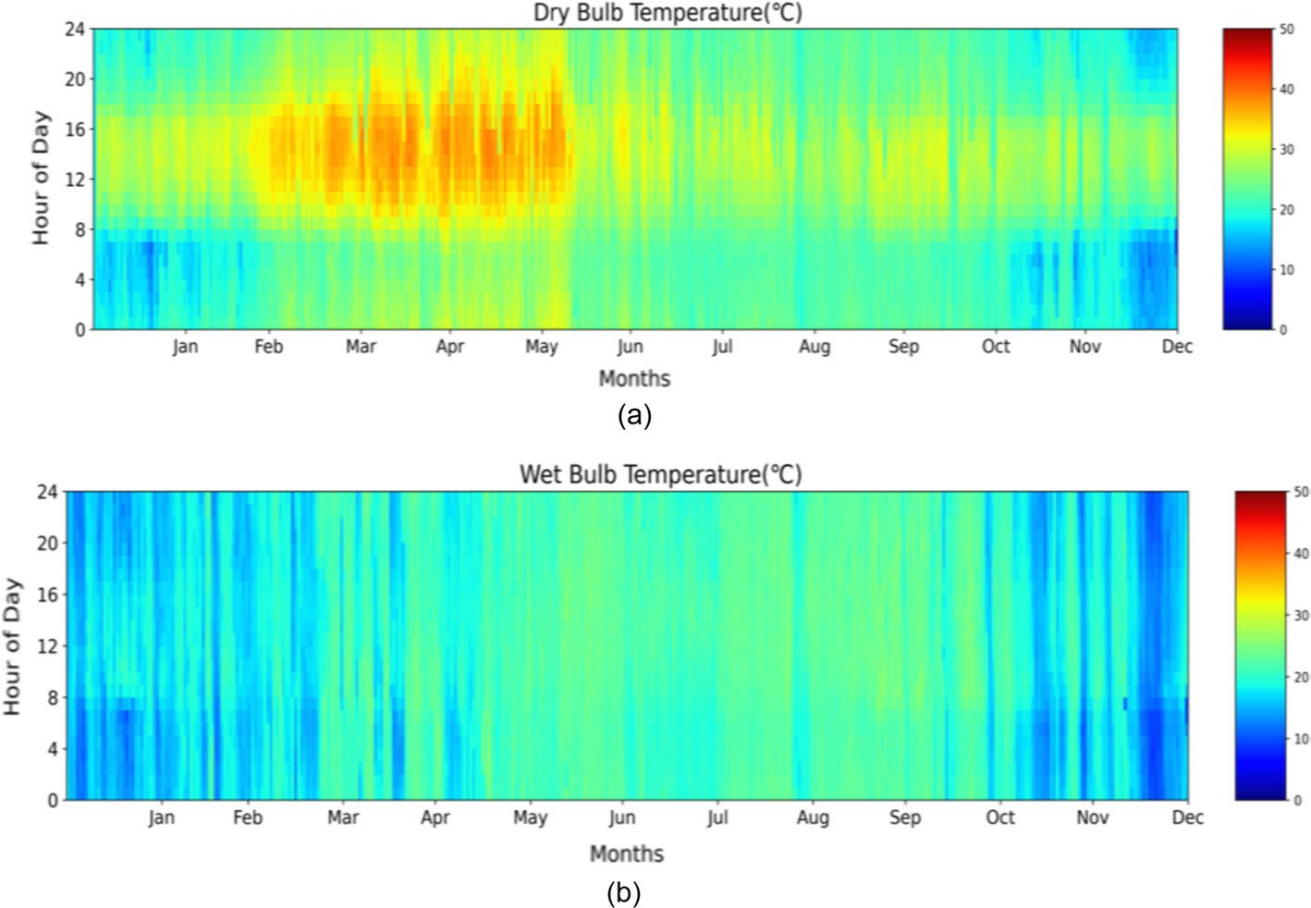
**a** Dry Bulb Temperature (°C) for Hyderabad **b** Wet Bulb Temperature (°C) for Hyderabad (ISHRAE Weather Data 2022)

The data utilized for this study was gathered from a residential complex in Hyderabad. It is a city in the Telangana region located in southern India. Hyderabad features an arid climate, where days are mostly dry and hot. The maximum average temperature can reach up to 40 °C (ISHRAE Weather Data 2022). It can be seen in Fig. 1a, b that the peak temperature in summer is during the months of April and May, which compels individuals to use AC. The year 2019 represents non-COVID year and 2020 represents COVID year. The lockdown in India was imposed on 25 March 2020. For our study, the months of April and May were found suitable. Since most people use AC during the summer season. To study only the effect of lockdown on the AC energy and household electricity consumption and keeping in mind the school summer vacation in May for children, the month of April was finally selected.

### Building and monitoring

**Fig. 2:**
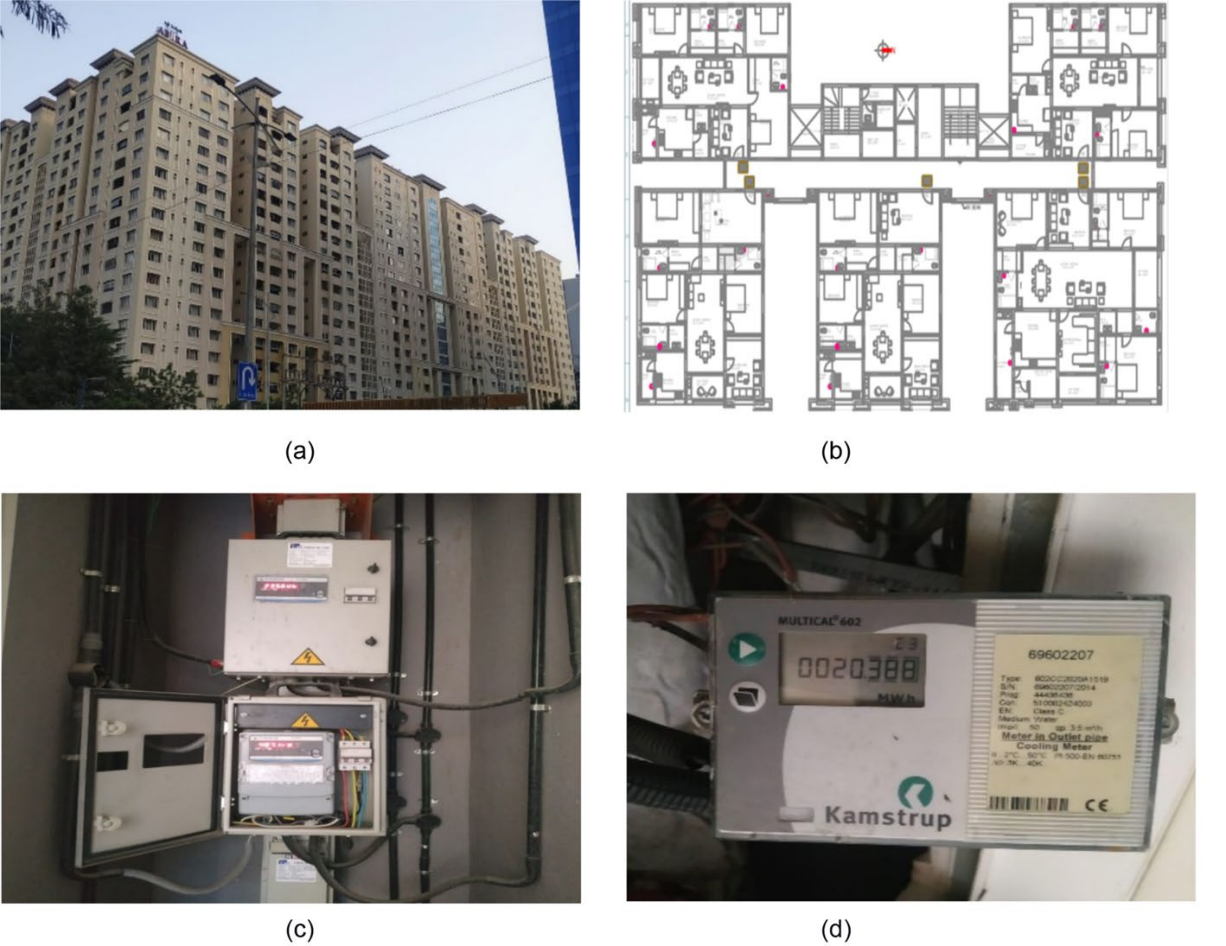
**a** Residential complex **b** Typical floor plan of the buildings **c** Energy meter **d** BTU meter

The pandemic COVID-19 was unforeseen and unpredicted, and luckily, we were able to collect the AC energy and electricity consumption data for the normal and COVID period. This type of data is rare, but we were able to collect the data from a residential complex consists of 380 homes in 5 buildings, 18 floors each, spread across 5 acres. The area of each home ranges from 180 to 450 sq.m, comprising 3 to 5 bedrooms, a living and drawing room, kitchen, and washrooms. All the rooms are facilitated by a central cooling system. For each home, household electricity was measured for the entire home except AC energy. Since chilled water system was used to provide cooling to the building, it was not part of the household electricity consumption. Only the power supplied to the fan of AC indoor unit was a part of household electricity consumption. The AC energy was measured separately in kWh thermal, and the household electricity was measured in kWh units. AC energy was measured using a metering system that records the cooling energy consumption by individual homes. There is an entity in the complex, which records electrical and cooling readings in 30 min interval separately. The electricity data of these homes were collected using smart meters, stored dynamically at the local database, and automatically retrieved every month and we received permission to use it for our study. These are commercial grid meters, and the readings were used for the billing purposes. Figure 2 consists of photographs of the residential complex, typical floor plan of the buildings, Energy meter and BTU meter along with its specifications.

### Data pre-processing

Thirty minute interval data (AC energy and household electricity consumption) for 380 homes was acquired for the month of April 2019 and 2020. In total 2,188,800 data points were obtained for this study. For this analysis weekdays were considered for each month from both the years, as the impact of lockdown can be better seen on working days. Using this criterion, we found 22 working days in April from each year.

The outdoor temperature for the Hyderabad region was collected from the website Visual crossing (https://www.visualcrossing.com/weather/weather-data-services#/viewData). It was observed that the average outdoor temperature for the year 2019 was more than that of 2020, as a result the energy consumption for 2020 was expected to be less (https://www.visualcrossing.com/weather/weather-data-services#/viewData). To overcome this, days with a similar average temperature range were selected out of the previously selected 22 days from both the years. We segregated the daily average temperatures in two sets, Set_lo for medium temperature range (31–33 °C) and Set_hi for high temperature range (33–35 °C). From the 22 selected days of each year, 7 days from 2019 and 6 days from 2020 were having average temperature ranging between 31 and 33 °C (Set_lo). For the temperature range of 33–35 °C there were 8 days from 2019 and 12 days from 2020 (Set_hi).

It was observed that for each set the average temperature in the days selected for 2019 and the days selected for 2020 were not similar. Further we wanted to ensure same number of days in both the years. We selected 5 days in each year in Set_lo, such that the average temperature in both the years was close to 32.05 °C. Similarly, 8 days were selected in each year in Set_hi, such that the average temperature in both the years was close to 33.92 °C. The analysis was performed on both the sets of temperature.

It was noticed that not all the 380 homes were occupied for the selected days. Further it was seen that some of the homes which were occupied during 2019 were not occupied during 2020 and vice-versa. To overcome this challenge, the common homes which were occupied for both years were taken to get more unbiased results. Two criteria were used to detect if the home was occupied: AC consumption to be greater than 1 kWh thermal and household electricity consumption greater than 1 kWh per day for the selected days. The common homes (between 2019 and 2020) qualifying both the criteria for all the selected days in the set were taken for the analysis. We filtered out 154 homes for Set_lo and 184 homes for Set_hi common in both the years. Table 1 provides the outcomes obtained of each filter.

**Table 1:** Details of filtration process

Filter number	Filter criteria	Number of days in 2019	Number of days in 2020	Average-temp 2019	Average-temp 2020	Number of homes 2019	Number of homes 2020
0	Raw data	30	30	33.20 °C	32.40 °C	380	380
1	Working days	22	22	33.14 °C	32.60 °C	380	380
2	Set_lo (medium temperature range)	7	6	31.95 °C	32.15 °C	380	380
	Set_hi (high temperature range)	8	12	33.93 °C	33.67 °C	380	380
3	Number of days further selected in Set_lo	5	5	32.06 °C	32.04 °C	380	380
	Number of days further selected in Set_hi	8	8	33.93 °C	33.91 °C	380	380
4	Common Occupancy in Set_lo	5	5	32.06 °C	32.04 °C	154	154
	Common Occupancy in Set_hi	8	8	33.93 °C	33.91 °C	184	184

By implementing this filtration process we ensure that each set has same number of working days and same homes in both the years with very close average temperature in both the years. The AC energy consumption, household electricity consumption and operating hours are calculated for the final filtered data that is shown in Table 1. The operating hours were calculated using the AC energy consumption data, if the AC energy consumption is zero for half-hour, we considered the AC to be off and vice-versa.

## Results

The average per day per home AC energy consumption and number of operating hours was calculated for both years in each set. Further, day and night AC consumption was also calculated. Similarly, average per day per home household energy consumption was calculated along with day and nighttime consumption. The results are tabulated along with the graphs for each analysis. The statistical tests for significance of the data points were also done and tabulated later in this section.

The average AC energy consumption per day per home for both the sets was calculated and analysed to find the energy consumed by the homes. It can be seen from Table 2 that the energy consumption for the year 2020 was 3.68% more for Set_lo and 4.40% more for Set_hi than year 2019. Further, to determine the daytime and nighttime AC energy consumption, AC energy data from 8:00 AM to 8:00 PM was taken to compute the daytime AC energy consumption. And the remaining data (8:00 PM to 8:00 AM) was taken to determine the nighttime AC energy consumption. It can be observed from Table 2, that during the lockdown period, the daytime consumption was high for both sets. The nighttime AC energy consumption for 2020 decreased by 3.65% for Set_lo and 5.11% for Set_hi when compared to 2019. This is expected to happen because of the pre-cooling effect in the homes, as the energy consumption for the year 2020 was more during the daytime than 2019.

**Table 2:** AC energy consumption, operating hours, and household electricity consumption for 2019 and 2020

	2019	2020	Net change (2020–2019)	Percentage Change (2020–2019/2019 *100)
Set_lo				
AC_EC_ Overall (kWh.th)	39.21	40.65	1.44	3.68
AC_EC_Day (kWh.th)	11.11	13.58	2.47	22.23
AC_EC_Night (kWh.th)	28.11	27.08	− 1.03	− 3.65
AC_OH_Overall (Hours)	10.06	10.82	0.76	7.55
AC_OH_Day (Hours)	2.77	3.82	1.05	37.91
AC_OH_Night (Hours)	7.29	7.00	− 0.29	− 3.98
HEC_Overall (kWh)	12.57	14.46	1.89	15.04
HEC_Day (kWh)	6.44	8.20	1.76	27.33
HEC_Night (kWh)	6.14	6.26	0.12	1.95
Set_hi				
AC_EC_Overall (kWh.th)	41.12	42.93	1.81	4.40
AC_EC_Day (kWh.th)	12.57	15.84	3.27	26.01
AC_EC_Night (kWh.th)	28.55	27.09	− 1.46	− 5.11
AC_OH_Overall (Hours)	10.66	11.19	0.53	4.97
AC_OH_Day (Hours)	3.23	4.16	0.93	28.79
AC_OH_Night (Hours)	7.45	7.05	− 0.40	− 5.37
HEC_Overall (kWh)	12.17	13.98	1.81	14.87
HEC_Day (kWh)	6.40	7.84	1.44	22.50
HEC_Night (kWh)	5.77	6.15	0.38	6.59

**EC = Energy consumption; OH = Operating hours; HEC = Household electricity consumption, Overall = for the complete day, Day = 8:00 AM to 8:00 PM, Night = 8:00 PM to 8:00 AM

Similarly, out of 48 half-hours, the average number of hours the AC was used per day per home for the two sets was determined. From Table 2, we can conclude that the AC was used more in 2020 as compared to 2019. On an average, AC was operated 11 h per day in 2020 as compared to 10 h in 2019. The daytime AC operating hours approximately increased by one hour for both the sets. The nighttime AC operating hours were similar for both the years; however, the nighttime average operating hours was less in 2020 by 3.98% for Set_lo and 5.37% for Set_hi when compared to 2019.

It can be observed that the AC energy consumption, and operational hours of the AC is more for Set_hi as compared to Set_lo for the same year. It can be concluded, because of the higher temperature range for Set_hi the consumption is more. It can also be seen that the percentage increase in AC energy consumption for Set_lo is much less than the percentage increase in number of operational hours, whereas for Set_hi they are similar.

**Fig. 3:**
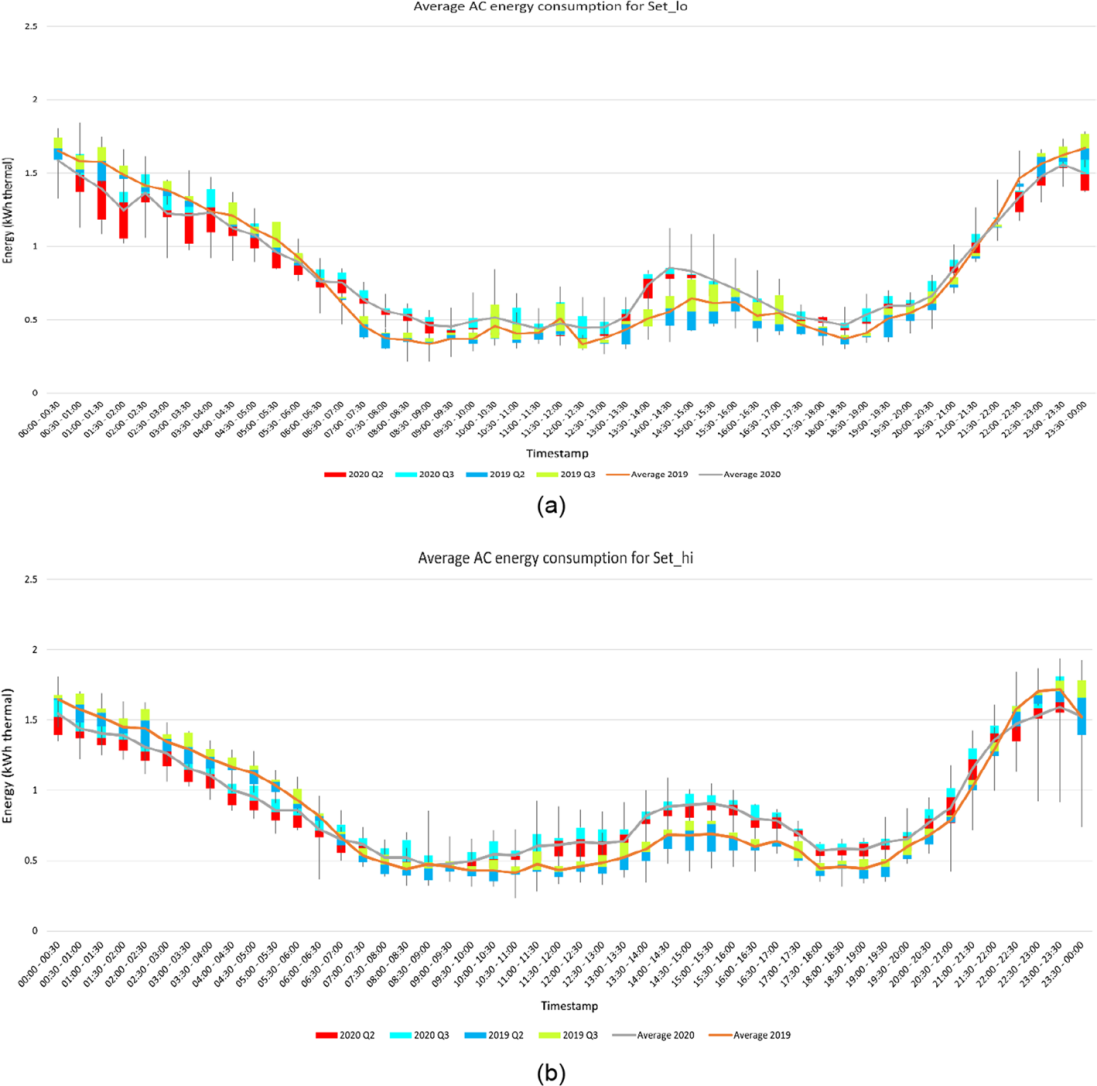
**a** AC energy consumption throughout the day for Set_lo **b** AC energy consumption throughout the day for Set_hi

**Fig. 4:**
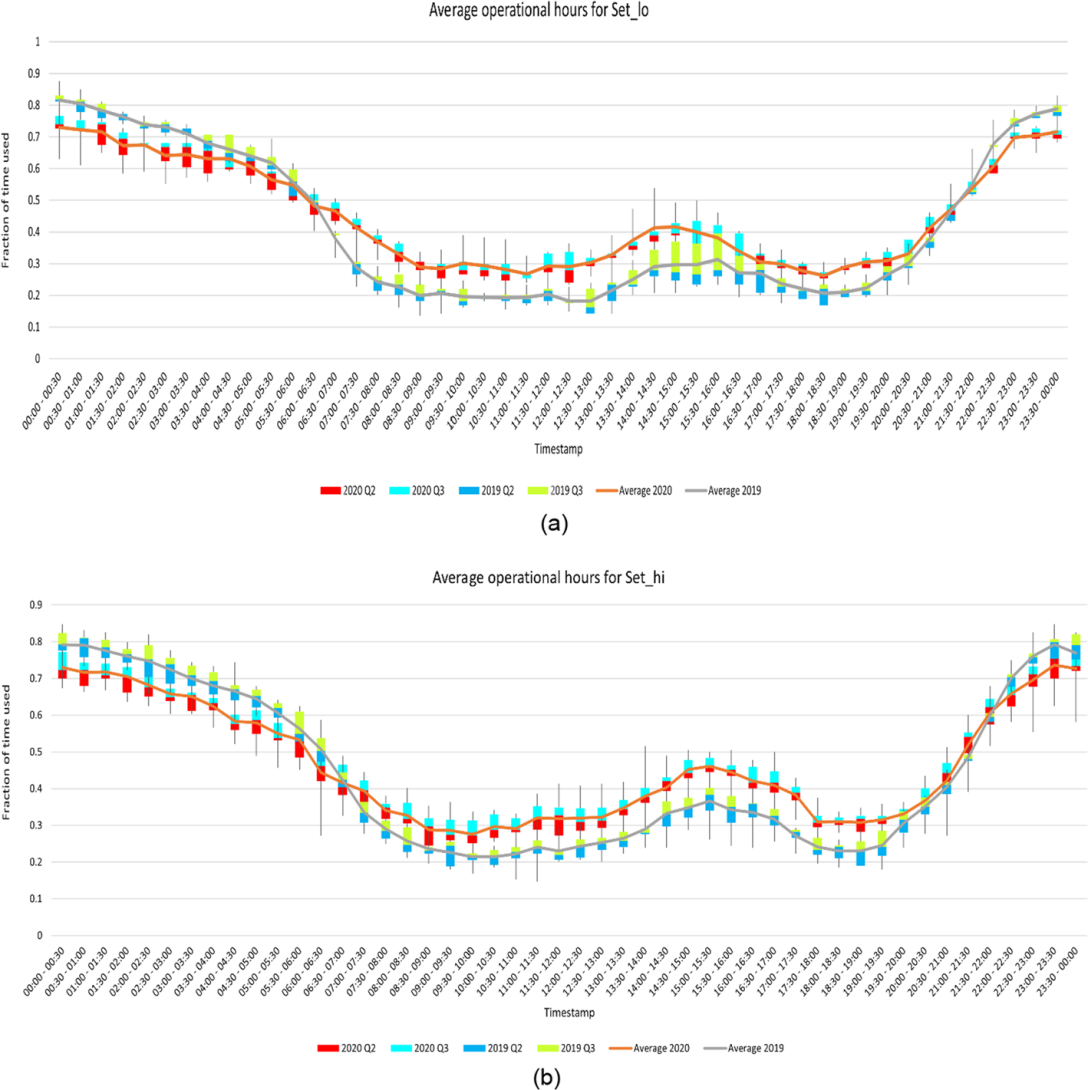
**a** Half-hour the AC was operated throughout the day for Set_lo **b** Half-hour the AC was operated throughout the day for Set_hi

From Table 2, it can be observed that the AC energy consumption for Set_lo has increased by 3.68% whereas the number of operational hours increased by 7.55%. Whereas the amount of increase in AC energy consumption, and the increase in operational hours for Set_hi is similar. This can be explained from the graphs in Fig. 3. The AC energy consumption for Set_hi is noticeably more after 9:00 AM, whereas AC energy consumption for Set_lo is more around 8:00 AM after which the energy consumption decreases till 12:00 PM. If we look at the operating hours graphs in Fig. 4, the AC was equally functional for both the sets. The temperature range for Set_lo is medium, due to the pre-cooling effect on the homes, the cooling load on the AC is less. However, for Set_hi, due to higher temperature the cooling load is higher, and the AC must consume more energy to reach the desired set point temperature provided by the occupants.

**Fig. 5:**
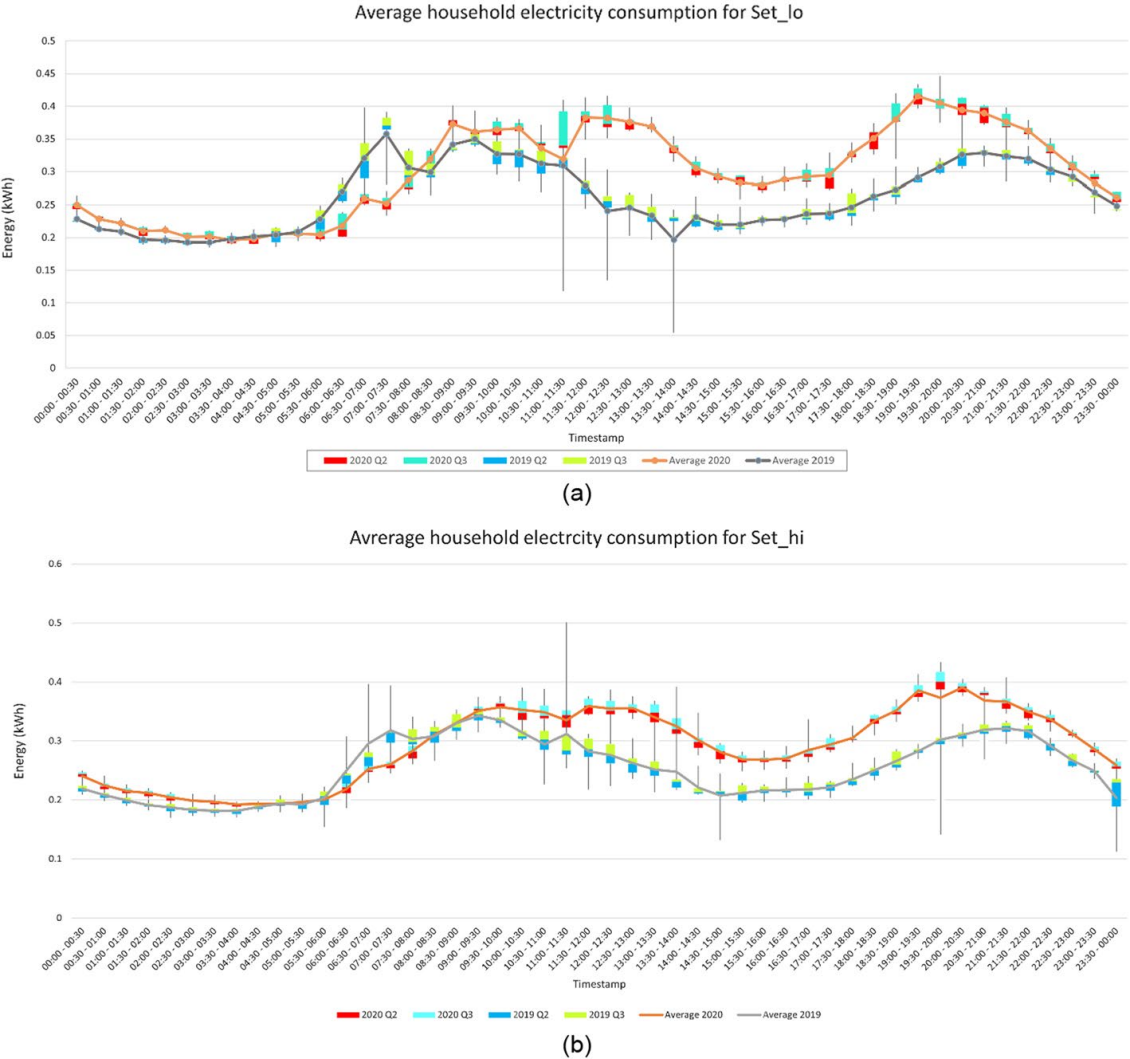
**a** Household electricity consumption throughout the day for Set_lo **b** Household electricity consumption throughout the day for Set_hi

The average per day per home household electricity consumption was also computed and plotted in Fig. 5. It can be seen in Table 2 that the household electricity consumption in the year 2020 increased by 15.04% for Set_lo and 14.87% for Set_hi than year 2019. The increment in household electricity consumption is similar for both the sets, as there is no direct impact of temperature on the household electricity consumption. Also, the percentage increase in household electricity consumption is significantly more during the day. Whereas the night household electricity consumption is similar in both the sets for both the years. Furthermore, the household electricity consumption for the year 2019 was more during the early morning between 4:00 AM to 8:00 AM. For the rest of the day the electricity was consumed more for the year 2020. These results indicate that people were using more electrical appliances such as monitors, laptops, TV, lights, cooking inductions during the lockdown as people were staying home.

### Statistical test for significance

The significance test is performed to check whether the observed differences between assessment results occur because of sampling error or chance. If the test turns out to be insignificant, then the results should not be considered because they do not reflect real differences (https://nces.ed.gov/nationsreportcard/NDEHelp/WebHelp/significance_tests_definition.htm). There are multiple significance tests in literature (https://home.csulb.edu/~msaintg/ppa696/696stsig.htm) among which, the t-test (https://www.scribbr.com/statistics/t-test/) was more suitable for our purposes. T-Tests are tests for statistical significance that are used with interval and ratio level data. It can be used for several different types of statistical tests, such as, to test whether there are differences between two groups on the same variable, based on the mean (average) value of that variable for each group, to test whether a group's mean (average) value is greater or less than some standard or to test whether the same group has different mean (average) scores on different variables (https://home.csulb.edu/~msaintg/ppa696/696stsig.htm). For our study paired t-tests were used, as the samples were from a single population (https://www.scribbr.com/statistics/t-test/). The test was conducted on the AC energy, number of hours AC was operated and electricity data as the data obtained is from the same source i.e., homes. The test was performed using a 95% confidence interval. Table 3 presents results obtained for the statistical test comparison of 2019 and 2020 for three sets of samples: AC energy, operating hours, and household electricity data. If the magnitude of t-stat value is less than t-critical two-tail, then we assume the null hypothesis is true. From Table 3 it can be seen that the t-stat values for all the 3 samples are greater than t-critical two-tail, hence the results provide strong evidence against the null hypothesis that μ2019 is equal to μ2020, hence concluding that “means of these two data sets for the same set of objects when compared pre-COVID and COVID years are statistically different for energy and number of operating hours.”

**Table 3:** Paired t-test analysis

Parameters	AC energy 2019	AC energy 2020	Operating hour 2019	Operating hour 2020	Household electricity 2019	Household electricity 2020
Mean	40.47	42.10	20.92	22.09	12.31	14.05
Variance	720.26	769.78	90.76	121.72	28.20	34.46
Number of observations	2242.00	2242.00	2242.00	2242.00	2242.00	2242.00
Degree of freedom	2241.00		2241.00		2241.00	
t-stat	− 3.04		− 5.31		− 20.81	
P (T ≤ t) two tail	0.002		12.4E-8		4.66E-88	
t-critical two tail	1.961		1.961		1.961	

## Discussion

Kawka and Cetin’s (2021) study on 225 homes for energy consumption during the lockdown period, observed that most of the energy consumption increase was between 10 AM and 1 PM. They also noticed a possibility of evening peak shifting earlier during the lockdown, assuming that home occupants complete their evening routines sooner than pre-pandemic periods without the commute. In our study, we observed that maximum energy consumption occurred during the day between 10 AM and 8 PM because people were working from home and probably woke up later in the morning. We went a step further and collected data for household electricity usage that showed how the evening peak shifted by approximately two hours during the lockdown period.

According to another study by Rouleau and Gosselin (2021), the occupants of residential buildings used more energy during lockdown when compared to non-COVID years. They observed that the average daily electricity consumption increased by 17.5% for April and between “9 AM to 5 PM”, the consumption increased by 46%. In comparison, as per our study, an increase of 24.5% was seen during a window of “8 AM to 8 PM”. According to Bielecki et al. study (Bielecki et al. 2021), the peak demand for 7000 apartments on average increased by about 9% during the lockdown compared to the same time of the year before the lockdown. Similarly, Aldubyan (2022) also observed a increase of 25.2% for residential buildings in Saudi Arabia. Sanchez-Lopez et al. (2022) also had a similar observation, as they found an increase of 17% in energy consumption. As per our analysis, the household electricity consumption increased by 15% for the samples collected from a residential complex in Hyderabad, India. Overall, we can see the residential energy consumption for households increased in several countries.

Given the peak load, Ahmed Abdeen (2021) reported an increase of peak load by 15–20% in the COVID year compared to the non-COVID year. Our study shows an increase upto 17–23% in household electricity consumption for April during the lockdown period. The variation in peak load results can be because of several factors like the climate of the place, income group of people from which the data was collected, occupancy, and building type.

The Prayas group (https://www.prayaspune.org/peg/blogs/household-electricity-consumption-in-indiaduring-the-covid-19-lockdown-insights-from-metering-data.html) obtained the electricity data from State Load Dispatch Centers (SLDCs) for Maharashtra and Uttar Pradesh from 21st March to 26th March 2020. According to them, the overall electricity consumption for Maharashtra decreased by 32%, and a reduction of 17% was seen for Uttar Pradesh when compared to previous year’s electricity consumption. According to them, residential electricity consumption contributes about 19% of the total electricity in Maharashtra, whereas it contributes 43% in Uttar Pradesh. According to their hypothesis, because of more contribution from the residential sector in Uttar Pradesh, the total energy consumption dropped less for Uttar Pradesh when compared to Maharashtra.

Furthermore, they analysed the household electricity for a few places in both states. They have analysed the metering data gathered from 81 households from both states. Their analysis period was from 4th March to 5th May 2020; however, the lockdown in India was from 25th March 2020. The daily average for the sample increased by 26% when compared to the lockdown period. According to them, the increase can be because of two factors: an increase in summer temperatures and people spending more time at home during the lockdown. But, according to our observation, the average temperature was more in 2020 than in 2019.

They have observed the households with AC in Pune district, and Pune city in Maharashtra increased by 45–60%. In contrast, there was an increase of 22% for households without AC. Further, they observed an increase of 35% and 45% for Aurangabad and Gonda, respectively.

In our study, there was an increase of 3–4% for AC energy consumption and 15% increase for household electricity consumption of the residential buildings in Hyderabad. We included a significant number of data points to provide more reliable results, such as analyzing with similar temperature and ensuring the analysis was done on homes occupied for both years.

## Conclusion

This paper explores the effect of lockdown due to COVID-19 on residential buildings. Due to the stay-at-home orders, the energy demands of residential buildings increased. Based on the hypotheses made and analysis performed, following are the key observations:

- The household electricity consumption increased during the lockdown which supports the first hypothesis. It can be explained as occupants worked from home, and used monitors, laptops, T.V, internet, lighting which in turn increased the household electricity consumption.
- The overall AC energy consumption and operational hours increased in 2020 compared to 2019. During daytime the AC energy consumption was more in 2020, but during the nighttime the consumption is more for 2019, hence the second hypothesis can be claimed to be true. Since the occupants were expected to operate the AC during the night after coming back from work in the year 2019, the nighttime energy consumption is more for 2019 than 2020. As the AC was functional throughout the day in 2020 the pre-cooling effect was observed since the set was already maintained.
- The AC energy consumption is more for Set_hi due to the higher temperature range. The effect of temperature is not seen in the household electricity consumption; both the sets have a similar increase of about 15%. Since there is no impact of temperature on the household electricity consumption.
- The peak in household electricity consumption for the year 2019 was seen in the morning, but for 2020 the peak shifted from morning to noon, supporting the third hypothesis. This indicates the occupants started waking up late, as they didn’t have to commute to their workplaces. Also, there is an earlier evening peak for 2020 than 2019, as occupants in 2019 used to come home after 6:00 PM from their workplaces.

COVID-19 made a significant impact on our lives due to which major adjustments were made. Some of these adjustments were of benefit e.g., office employees saved significant time, stress, and money by working from home, instead of commuting by private or public transport. As the world becomes increasingly technology driven, workplace policies may change and work from home may increase. Based on these inferences, energy consumption of commercial and residential buildings can be analysed to understand the potential effects and steps can be taken accordingly. For example, we observed that the peak electricity consumption during lockdown has increased during the day in residential sectors, moreover there was an occurrence of an additional peak. Insights of this nature can help with planning the development of appropriate infrastructure, such as transformers and power plants. If similar studies conducted on a larger number of samples result in similar observations, energy policies can be designed at national level for a more efficient usage of energy.

## Data Availability

The datasets generated and/or analysed during the current study are not publicly available as more research works are in progress with the same data but are available from the corresponding author on reasonable request.

## Abbreviations

AC: Air condition
EC: Energy consumption
OH: Operating hours
HEC: Household electricity consumption
EC: Energy consumption
OH: Operating hours
HEC: Household electricity consumption
Overall: For the complete day
Day: 8:00 AM to 8:00 PM
Night: 8:00 PM to 8:00 AM

## References

[CR1] Abdeen A, Kharvari F, O’Brien W, Gunay B (2021). The impact of the COVID-19 on households’ hourly electricity consumption in Canada. Energy Build.

[CR2] About India at a glance- IBEF. https://www.ibef.org/economy/indiasnapshot/aboutindia-at-a-glance Accessed 4 Apr 2022

[CR3] Alavi A, Sadid MS, Ahmed M, Abid F (2022). Effect analysis of the COVID-19 pandemic on the electricity consumption of Bangladesh. Heliyon.

[CR4] Aldubyan M, Krarti M (2022). Impact of stay home living on energy demand of residential buildings: Saudi Arabian case study. Energy.

[CR5] Andrews M, Areekal B, Rajesh K, Krishnan J, Suryakala R, Krishnan B, Muraly C, Santhosh P (2020). First confirmed case of COVID-19 infection in India: a case report. Indian J Med Res.

[CR6] Ashkanani AM, Bahman AM, Aljuwayhel NF (2022). Impact of COVID-19 interventions on electricity power production: an empirical investigation in Kuwait. Electric Power Syst Res.

[CR7] Bielecki S, Dukat P, Skoczkowski T, Sobczak L, Buchoski J, Maciag Ł (2021). Impact of the lockdown during the covid-19 pandemic on electricity use by residential users. Energies (basel).

[CR8] Burleyson CD, Rahman A, Rice JS, Smith AD, Voisin N (2021). Multiscale effects masked the impact of the COVID-19 pandemic on electricity demand in the United States. Appl Energy.

[CR9] Chen C, Zarazua de Rubens G, Xu X, Li J (2020). Coronavirus comes home? Energy use, home energy management, and the social-psychological factors of COVID-19. Energy Res Soc Sci.

[CR10] Chinthavali S, Tansakul V, Lee S (2022). COVID-19 pandemic ramifications on residential Smart homes energy use load profiles. Energy Build.

[CR11] Coronavirus disease (COVID-19)—World Health Organization. https://www.who.int/docs/default-source/coronaviruse/situation-reports/20200327-sitrep-67-covid-19.pdf. Accessed 2 Mar 2022

[CR12] COVID-19 impact on electricity- IEA. https://www.iea.org/reports/covid-19-impacton-electricity. Accessed 10 Feb 2022

[CR13] COVID-19 restrictions changing the daily patterns of energy consumption. https://www.savills.us/insight-and-opinion/savills-news/299070/covid-19-restrictions-changing-the-daily-patterns-of-energy-consumption. Accessed 7 Feb 2022

[CR14] Ding Y, Ivanko D, Cao G, Brattebø H, Nord N (2021). Analysis of electricity use and economic impacts for buildings with electric heating under lockdown conditions: examples for educational buildings and residential buildings in Norway. Sustain Cities Soc.

[CR15] Edomah N, Ndulue G (2020). Energy transition in a lockdown: an analysis of the impact of COVID-19 on changes in electricity demand in Lagos Nigeria. Global Transitions.

[CR16] Global Energy Review 2020. https://www.iea.org/reports/global-energy-review2020. Accessed 8 Mar 2022

[CR17] Guidelines on the measures to be taken by Ministries/ Departments of Government of India, State/ Union Territory Governments and State/ Union Territory Authorities for containment of COVID-19 Epidemic in the Country. https://www.mha.gov.in/sites/default/files/Guidelines_0.pdf. Accessed 28 Feb 2022

[CR18] H. Osbourne Austin’s coronavirus stay-home order could swell utility bills, Austin Am.-Statesman. https://www.statesman.com/story/news/local/flashbriefing/2020/03/25/austins-coronavirus-stay-home-order-could-swell-utilitybills/1459345007/ Accessed 15 Feb 2022

[CR19] Household electricity consumption during the COVID-19 lockdown. https://www.prayaspune.org/peg/blogs/household-electricity-consumption-in-indiaduring-the-covid-19-lockdown-insights-from-metering-data.html Accessed 4 Feb 2022

[CR20] How technology changed lives during COVID-19 lockdown. https://zeenews.india.com/technology/how-technology-changed-lives-during-covid19-lockdown-2349849.html. Accessed 5 Mar 2022

[CR21] Huebner GM, Watson NE, Direk K, McKenna E, Webborn E, Hollick F, Elam S, Oreszczyn T (2021). Survey study on energy use in UK homes during COVID-19. Build Cities.

[CR22] India Situation Report—WHO. https://www.who.int/india/emergencies/coronavirusdisease-(covid-19)/india-situation-report. Accessed 27 Feb 2022

[CR23] India’s decline in electricity consumption due to lockdown more severe than US and EU: Study. https://www.indiatoday.in/india/story/india-s-decline-in-electricityconsumption-due-to-lockdown-more-severe-than-us-and-eu-1666444-2020-04-13 Accessed 18 Feb 2022

[CR24] ISHRAE Weather Data 2022. https://shop.ishrae.in/product/details/e-bookweather-data-/86 Accessed 14 Feb 2022

[CR25] Kawka E, Cetin K (2021). Impacts of COVID-19 on residential building energy use and performance. Build Environ.

[CR26] Krarti M, Aldubyan M (2021). Review analysis of COVID-19 impact on electricity demand for residential buildings. Renew Sustain Energy Rev.

[CR27] Ku AL, Qiu Y, Lou J, Nock D, Xing B (2022). Changes in hourly electricity consumption under COVID mandates: a glance to future hourly residential power consumption pattern with remote work in Arizona. Appl Energy.

[CR28] Li L, Meinrenken CJ, Modi V, Culligan PJ (2021). Impacts of COVID-19 related stay-at-home restrictions on residential electricity use and implications for future grid stability. Energy Build.

[CR29] March 25, 2020: The day India went into nationwide lockdown to tackle coronavirus. https://www.timesnownews.com/india/article/march-25-2020-the-dayindia-went-into-nationwide-lockdown-to-tackle-coronavirus/736838. Accessed 27 Feb 2022

[CR30] Portugal 2021- iea. https://www.iea.org/reports/portugal-2021. Accessed 13 Feb 2022

[CR31] Qarnain SS, Sattanathan M, Sankaranarayanan B, Ali SM (2020). Analyzing energy consumption factors during coronavirus (COVID-19) pandemic outbreak a case study of residential society. Energy Sources Part A Recovery Util Environ Eff.

[CR32] Rebecca Bevans an introduction to T-Tests | Definitions, Formula and Examples- Scribbr. https://www.scribbr.com/statistics/t-test/. Accessed 3 Feb 2022

[CR33] Rouleau J, Gosselin L (2021). Impacts of the COVID-19 lockdown on energy consumption in a Canadian social housing building. Appl Energy.

[CR34] Sánchez-López M, Moreno R, Alvarado D, Suazo-Martínez C, Negrete-Pincetic M, Olivares D, Sepúlveda C, Otárola H, Basso LJ (2022). The diverse impacts of COVID-19 on electricity demand: the case of Chile. Int J Electr Power Energy Syst.

[CR35] Sánchez-Úbeda EF, Portela J, Muñoz A, Chueca Montuenga E, Hallack M (2022). Impact of COVID-19 on electricity demand of Latin America and the Caribbean countries. Sustain Energy Grids Netw.

[CR36] Scott Hinson COVID-19 is Changing Residential Electricity Demand- TD World. https://www.tdworld.com/distributed-energy-resources/demand-sidemanagement/article/21128542/covid19-is-changing-residential-electricity-demand. Accessed 5 Mar 2022

[CR37] Significance Tests: Definition. https://nces.ed.gov/nationsreportcard/NDEHelp/WebHelp/significance_tests_definition.htm. Accessed 9 Feb 2022

[CR38] Snow S, Bean R, Glencross M, Horrocks N (2020). Drivers behind residential electricity demand fluctuations due to COVID-19 restrictions. Energies (basel).

[CR39] Summers in India. https://www.indiaonlinepages.com/weather/summers-in-india.html. Accessed 8 Feb 2022

[CR40] Surahman U, Hartono D, Setyowati E, Jurizat A (2022). Investigation on household energy consumption of urban residential buildings in major cities of Indonesia during COVID-19 pandemic. Energy Build.

[CR41] Test for Significance- California State University. https://home.csulb.edu/~msaintg/ppa696/696stsig.htm. Accessed 9 Feb 2022

[CR42] Weather Query Builder. https://www.visualcrossing.com/weather/weather-data-services#/viewData. Accessed 8 Feb 2022

[CR43] WeatherOnline. https://www.weatheronline.co.uk/reports/climate/India.htm Accessed 4 Mar 2022

[CR44] Wen L, Sharp B, Suomalainen K, Sheng MS, Guang F (2022). The impact of COVID-19 containment measures on changes in electricity demand. Sustain Energy Grids Netw.

[CR45] Worldometer. https://www.worldometers.info/coronavirus/country/india/. Accessed 5 Mar 2022

[CR46] Yukseltan E, Kok A, Yucekaya A, Bilge A, Aktunc EA, Hekimoglu M (2022). The impact of the COVID-19 pandemic and behavioral restrictions on electricity consumption and the daily demand curve in Turkey. Util Policy.

